# Dual-Task Performance in Hearing-Impaired Older Adults— Study Protocol for a Cross-Sectional Mobile Brain/Body Imaging Study

**DOI:** 10.3389/fnagi.2021.773287

**Published:** 2021-11-12

**Authors:** Anna Wunderlich, Oliver Vogel, Maja Maša Šömen, Manca Peskar, Madeleine Fricke, Klaus Gramann, Janna Protzak, Uros Marusic, Bettina Wollesen

**Affiliations:** ^1^Biological Psychology and Neuroergonomics, Department of Psychology and Ergonomics, Faculty V: Mechanical Engineering and Transport Systems, Technische Universität Berlin, Berlin, Germany; ^2^Human Movement and Training Science, Institute of Human Movement Science, Psychology and Human Movement, University Hamburg, Hamburg, Germany; ^3^Science and Research Centre Koper, Institute for Kinesiology Research, Koper, Slovenia; ^4^Department of Health Sciences, Alma Mater Europaea – ECM, Maribor, Slovenia

**Keywords:** hearing impairment, MoBI, dual-task, overground walking, older adults

## Abstract

**Background:** Hearing impairments are associated with reduced walking performance under Dual-task (DT) conditions. Little is known about the neural representation of DT performance while walking in this target group compared to healthy controls or younger adults. Therefore, utilizing the Mobile Brain/Body Imaging approach (MoBI), we aim at gaining deeper insights into the brain dynamics underlying the interaction of cognitive and motor processes during different DT conditions (visual and auditory) controlling for age and the potential performance decrements of older adults with hearing impairments.

**Methods:** The cross-sectional study integrates a multifactorial mixed-measure design. Between-subject factors grouping the sample will be age (younger vs. older adults) and hearing impairment (mild vs. not hearing impaired). The within-subject factors will be the task complexity (single- vs. DT) and cognitive task modality (visual vs. auditory). Stimuli of the cognitive task will vary according to the stimulus modality (visual vs. auditory), presentation side (left vs. right), and presentation-response compatibility (ipsilateral vs. contralateral). Analyses of DT costs and underlying neuronal correlates focus either on gait or cognitive performance. Based on an *a priori* sample size calculation 96 (48 healthy and 48 mildly hearing impaired) community-dwelling older adults (50–70 years) and 48 younger adults (20–30 years) will be recruited. Gait parameters of speed and rhythm will be captured. EEG activity will be recorded using 64 active electrodes.

**Discussion:** The study evaluates cognitive-motor interference (CMI) in groups of young and older adults as well as older adults with hearing impairment. The underlying processes of the interaction between motor and cognitive tasks will be identified at a behavioral and neurophysiological level comparing an auditory or a visual secondary task. We assume that performance differences are linked to different cognitive-motor processes, i.e., stimulus input, resource allocation, and movement execution. Moreover, for the different DT conditions (auditory vs. visual) we assume performance decrements within the auditory condition, especially for older, hearing-impaired adults. Findings will provide evidence of general mechanisms of CMI (ST vs. DT walking) as well as task-specific effects in dual-task performance while over ground walking.

## Introduction

Age-related decline in hearing is one of the most common chronic conditions in older adults, affecting nearly half of people over the age of 65 (Goman and Lin, 2016). In recent decades, a worldwide increase in the prevalence of hearing impairments has been observed. With 42 million people affected worldwide in 1985 (∼0.8%), the number increased more than eightfold to 360 million in 2011 (∼5.2%, Olusanya et al., 2014; Olusanya et al., 2019). According to current research, this number grew further to 466 million people (∼6.1%) in 2018 and is estimated to increase to 900 million in 2050 (World Health Organization, 2020). These numbers emphasize the increasing importance of hearing impairment as a public health burden.

Longitudinal studies have shown that hearing impairment is independently associated with poorer cognitive performance even when controlling for age and sex (Valentijn et al., 2005; Lin, 2011). Several researchers have tried to explain this association between age-related hearing impairment and cognitive decline (cf. Mudar and Husain, 2016). Some have proposed a common cause hypothesis, which explains hearing impairment and cognitive decline as a widespread neural degeneration that occurs during aging. Others have proposed an information degradation hypothesis, which suggests poorer cognitive performance as a result of hearing impairment. Presumably, additional cognitive resources are devoted to auditory processing, resulting in fewer resources available for other processes. The sensory deprivation hypothesis views poorer cognitive performance not as an instantaneous consequence of the hearing impairment as the information degradation hypothesis does, but as a long-term change in brain plasticity (Mudar and Husain, 2016).

Age-related changes in the auditory system can include both, higher pure-tone detection thresholds and supra-threshold auditory difficulties (Schneider et al., 2010), requiring a more deliberate allocation of resources to hearing. Consequently, hearing becomes more effortful and cognitively demanding (Pichora-Fuller et al., 2016). Beyond hearing difficulties, hearing impairment further affects physical activities (Chen et al., 2014; Gispen et al., 2014). As a result of cognitive and physical challenges, hearing impairment negatively affects several aspects of one’s personal and social life, such as psychosocial well-being, quality of life, economic independence, and interpersonal communication (Mick et al., 2014). Frequently observed consequences encompass social isolation and stigmatization, substance abuse, psychiatric disturbance, depression, difficulties in relationships with partners and children as well as occupational stress (Olusanya et al., 2019). Moreover, activities of daily living (ADL) are further aggravated by hearing impairments in older adults (Chang et al., 2009; Gopinath et al., 2016). Effects of age-related hearing impairment on locomotion and cognition were examined in comparison to age-matched reference groups with hearing-impaired older adults exhibiting lower levels of physical activity and functional performance (Chen et al., 2014; Gispen et al., 2014). A decline in hearing has been found to correlate with balance impairments (Viljanen et al., 2009a,b), self-reported walking limitations (Chen et al., 2014), poor endurance (Gopinath et al., 2016), and increased frailty (Kamil et al., 2014). There are also findings on gait parameters that accompany hearing impairment. An increasing deficit of auditory perception has been found to negatively affect stride length as well as gait speed and cadence under dual-task conditions, independent of age and comorbidities (Wollesen et al., 2018). The effects of hearing impairment on gait even exceed the influence of age, disease, and previous falls (Lin and Ferrucci, 2012). Furthermore, both hearing impairment and decrements in gait quality are associated with the risk of falling in older adults (Lin and Ferrucci, 2012; Jiam et al., 2016).

Researchers have attempted to explain the association between hearing impairment and mobility decline in terms of competition for limited shared cognitive resources (Bruce et al., 2019). In everyday life, balancing or walking is rarely the only task performed at a time (single-task) and it is mostly performed together with another task (dual-task) or sometimes even as a multitasking activity (Faulkner et al., 2007). Cognitive-motor dual-task (CMDT) studies usually compare a single-task condition, such as a postural or walking task, with a dual-task condition, which combines the same motor task with a synchronous cognitive task. By comparing performance in both conditions, we can then calculate dual-task costs (DTCs: single-task minus dual-task performance divided by single-task performance) which indicate the degree of performance decline in one or both tasks due to limited cognitive capacity (Li and Lindenberger, 2002; Marusic et al., 2015; Janouch et al., 2018; Wollesen et al., 2018). CMDT studies have shown that as cognitive load increases during the manipulation of sensory information, older adults show greater postural performance decrements compared to their younger counterparts (Redfern et al., 2001; Doumas et al., 2008). Competition for cognitive capacity is also observed when auditory challenges are experimentally imposed on different motor tasks, such as balancing or walking (Nieborowska et al., 2019), or when older adults with hearing impairment undergo CMDT (Lau et al., 2016). The so-called “posture-first” hypothesis or strategy (Shumway-Cook et al., 1997) describes the prioritization of physical safety by allocating more attention to motor performance than to cognitive performance. In older adults, such prioritization increases with task complexity, postural threat, or fear of falling (Li et al., 2001).

Since older adults often exhibit hearing impairments that severely affect their lives, investigating the interaction between age-related hearing impairment and decline in other domains (e.g., vision, cognition, and mobility) at the behavioral and neurophysiological levels could elucidate the underlying mechanisms. In addition, this research could lead to therapeutically promising insights by providing a better understanding of the risk of falls leading toward improved fall prevention programs.

Daily dual-task situations entail different levels of cognitive complexity and therefore will require different cognitive abilities. According to Colcombe and Kramer (2003), cognitive complexity can be classified as (1) simple stimulus-response reaction reflecting processing speed (e.g., reacting to stop at a red traffic light); (2) visuo-spatial tasks to orient in the environment (e.g., walking on uneven ground or avoiding puddles, detecting and deciding whether to evade an approaching object or person), and (3) executive tasks which represent action planning, and response-inhibition (e.g., crossing a street without traffic lights while constantly adapting movement to current traffic flow or remembering a shopping list). For all these daily activities visual information processing is ubiquitous and is relevant to walking itself (Rosano et al., 2012; Mahoney and Verghese, 2018; Owsley et al., 2018). Therefore, a visual dual-task condition while walking will be used as one experimental manipulation of this study. Furthermore, growing evidence indicates that hearing has an important influence on walking performance as well (Chang et al., 2009; Viljanen et al., 2009a; Gopinath et al., 2016; Wollesen et al., 2018). Moreover, daily situations requiring executive decision-making are often solved by a multi-sensory integration including hearing (e.g., the sound of approaching cars) and not only by vision (Wollesen et al., 2021). Therefore, this study will also look at neuronal correlates of dual-task walking with an auditory secondary task. As we assume that participants with hearing impairments compared to participants with normal hearing might have more difficulties during auditory dual-task walking situations, the study will allow for more insights about possible differences in cognitive processing (i) in the different dual-task situations and (ii) between the participants with and without hearing impairments.

Previous research on cognitive-motor interference (CMI) while dual-task walking showed that spatio-temporal gait parameters like walking speed, step length, and arm swing symmetry are affected by the secondary task (Mirelman et al., 2015; Killeen et al., 2017). Moreover, there is evidence that different pre-conditions of the individuals’ cognitive-motor abilities, such as balance decline, concern about falling, and hearing impairments, negatively influence dual-task walking, that is reduced walking speed, step length, heel-strike, and foot-rolling movements (Wollesen et al., 2017a,b). These effects were maintained after controlling for age and comorbidities and showed that older adults with concerns about falling or hearing impairment have worse initial conditions on their gait quality and therefore have an increased risk of falling. More research is warranted to confirm these findings, as they relied purely on behavioral data with biomechanical walking measurements and cognitive performance through error counting or reaction times. Therefore, it is relevant to investigate the interaction between the motor and the cognitive system by analyzing the processes and conditions that influence older adults’ gait performance in situations that require concurrent performance of sensorimotor and cognitive tasks.

The following experiment will use walking tasks with different complexities and priorities, and record behavioral (spatio-temporal gait parameters) as well as neurophysiological data.

### Mobile Brain/Body Imaging to Evaluate Cognitive-Motor Interference

Mobile brain/body imaging (MoBI; Makeig et al., 2009; Gramann et al., 2011, 2014) combines mobile brain imaging (in most cases Electroencephalography; EEG) of freely moving participants with synchronized recordings of task performance and body movements. MoBI is the only brain imaging method that allows for investigating the neuronal correlates of dual-task costs during over ground walking. Motion capture provides the necessary biomechanical data as well as measures of gait quality including arm swing and hip rotation. Walking is associated with movement of the head and the eyes producing electrical activity that will, due to volume conduction, be recorded as a mixture with other active sources in the brain at the sensor level (Jung et al., 2000). Recording and analyzing EEG data from actively walking participants thus requires analysis approaches that differentiate between the brain and non-brain activity. It was demonstrated that brain dynamics can be recorded and successfully analyzed using independent component analysis (ICA) even when subjects walk or run on a treadmill (Gramann et al., 2010; Gwin et al., 2010, 2011; Seeber et al., 2013; Wagner et al., 2013, 2014). Importantly, event-related spectral perturbations (ERSPs) demonstrated a desynchronization of theta oscillation when transitioning from standing to walking while the P300 event-related potential (ERP) component revealed amplitude reductions while walking (Wagner et al., 2016). A similar reduction of an ERP component amplitude was found for the N2 in a Go/NoGo task for dual-task walking as compared to sitting with older adults revealing less pronounced component amplitudes and increased latencies compared to young adults reflecting increased dual-task costs during walking (Malcolm et al., 2015). These behavioral and brain dynamic results indicate that walking poses higher attentional demands compared to standing and thus leaves fewer resources during walking for solving a secondary task. This effect is even more detrimental for older adults with an increased difficulty to allocate attention selectively across several domains (Protzak and Gramann, 2021). Age-related sensory and cognitive decline leads to an increase in demand on attention allocation and working-memory. This was for example shown in a speech in noise perception task by Wong et al. (2009) using fMRI investigating healthy older adults.

### Previous Neurophysiological Studies in Hearing Impaired

Evidence indicates several anatomical and functional brain alterations in hearing-impaired older adults. These changes occur not only in regions involved in auditory processing but also in regions involved in attention and emotional processing (cf. Mudar and Husain, 2016). For the present study, we will further describe the brain activity alterations in individuals with hearing impairment.

Two studies by Campbell and Sharma examined auditory (Campbell and Sharma, 2013) and visual evoked potentials (Campbell and Sharma, 2014) in adults with bilateral mild to moderate high-frequency (2–8 kHz) hearing loss and normal-hearing controls. In the auditory evoked potentials study (2013), performance on a speech-in-noise test was positively correlated with increased latencies of the frontal P2 component. Compared to the control group, the hearing-impaired participants showed increases in P2 latency and amplitude. Increased activation in the frontal cortex and decreased activation in the temporal cortex in hearing-impaired subjects compared to the control group were found using cortical source localization, indicating possible changes in the allocation of cortical resources. In the study published in 2014, Campbell and Sharma examined visual evoked potentials and correlated auditory performance in a speech perception task with the visual evoked N1 latency for persons with mild-moderate hearing loss. Adults with hearing loss showed decreased N1 latencies compared to controls and a negative correlation with auditory performance. Furthermore, the amplitudes of P1, N1, and P2 were significantly larger for hearing-impaired participants (Campbell and Sharma, 2014). Using source localization, the authors showed that the P1 component originated from similar brain areas (cerebellar and higher-order visual cortical regions) for both, hearing-impaired and healthy participants. However, for the N1 and P2 components, the hearing-impaired group showed increased activation in temporal areas, which are associated with auditory processing (e.g., superior temporal gyrus, medial temporal gyrus, and inferior temporal gyrus; Campbell and Sharma, 2014). This suggests visual cross-modal reorganization (i.e., intact visual systems can recruit and repurpose deprived audio cortices for processing of their input). Similarly, Cardon and Sharma (2018) investigated cross-modal reorganization between the auditory and somatosensory modalities in older adults with normal hearing and mild-moderate hearing in response to vibrotactile stimulation using high-density electroencephalography. Results showed activation of the somatosensory areas in both hearing-impaired and adults with normal hearing. However, adults with age-related hearing impairment also showed activation of auditory cortical regions in response to somatosensory stimulation.

Regarding the current literature, existing studies with hearing impaired older adults only addressed one of the described aspects but not the comparison of the two most relevant domains for daily activities (vision and hearing) for this target group. Moreover, the neural correlates of existing studies with hearing impaired participants were only examined in a sitting condition. It remains unclear if these findings can be transferred to daily situations that reflect dual-tasking aspects during real life activities. Therefore, the multicomponent MoBI-approach will help to overcome the research gap and gain deeper insights into the neural underpinnings of the interaction of motor and cognitive tasks in different dual-task conditions (visual and auditory). We will further systematically control for the impact of age and potential performance decrements of older adults with hearing impairments.

### Aims and Research Questions for the Study

The present study is designed to investigate the following three foci using a MoBI approach:

Focus 1: General characteristics of dual-task walking of all participants.

• How does cognitive task performance differ when using visual or auditory stimuli in single- and dual-task conditions?
• How are dual-task costs represented in gait parameters?
• How are neuronal correlates of auditory and visual information processing impacted by dual-task interference during walking?

Focus 2: Age-related differences in dual-task walking.

• Can we replicate the age-related increase in dual-task costs?
• How are the age-related differences in performance reflected in the respective brain activity of younger and older adults?
• How do younger and older adults differ in their gait parameters and gait-phase related stimuli processing?

Focus 3: Differences of hearing-impaired and healthy older participants in dual-task walking.

• How do auditory vs. visual information processing and movement control vary with regards to the effect of hearing impairment represented in both cognitive task stimuli modalities and dual-task vs. single-task performance?
• How are dual-task costs reflected in biomechanical and neuropsychological measures of hearing-impaired participants?

The overall hypothesis is that there are differences in dual-task costs and their corresponding neuronal correlates between the three groups with the highest disadvantages for the group of older adults with impaired hearing. We hypothesize that the performance differences are linked to the different cognitive-motor processes; i.e., information or stimulus input, resource allocation, and movement execution. Further, we hypothesize that the increasing task complexity (from single-task to dual-task), as well as the stimulus modality (visual vs. auditory), will further diminish the walking performance and increase neuronal activity.

## Methods/Study Design

This protocol paper was drafted according to the SPIRIT statement (Chan et al., 2013).

### Trial Design

This protocol will reflect a multifactorial mixed-measure design with the two two-level between-subject factors, *age* (younger vs. older adults) and *hearing impairment* (mild hearing-impaired vs. not hearing impaired). The design will be incomplete as the sample contains older adults with hearing impairments and healthy controls matched by age and gender as well as a younger age group. No data of young hearing-impaired adults will be recorded.

The within-subject factors will be the *task condition* (single- vs. dual-task) and *cognitive task modality* (visual vs. auditory). Stimuli of the cognitive task will vary according to the *presentation side* (left vs. right), *modality-specific properties* (magenta vs. cyan or low vs. high pitch, respectively), and *presentation-response compatibility* (ipsilateral vs. contra-lateral). Analyses of dual-task costs and underlying neuronal correlates can focus either on gait parameters or cognitive task performance.

The chosen colors and tone pitch levels are the conclusion of extensive piloting. In case further testing reveals that other properties are more appropriate for the experimental paradigm, we keep the option open to change the modality-specific properties.

### Participants, Interventions, and Outcomes

#### Ethical Approval

The study will be conducted in agreement with the principles of the Declaration of Helsinki and the guidelines of Good Clinical Practice. Written informed consent will be obtained from all participants before enrolment in the study. The local ethics committee of the TU Berlin, Germany, has approved the study protocol {BPN_WOL_1_210129}. The trial was registered at DRKS.de with registration number DRKS00024453 on April 14th, 2021.

#### Recruitment of Participants

To assure eligibility and recruitment of participants, community-dwelling older adults will be recruited from a database of the TU Berlin (e.g., “TUB-Versuchspersonenportal”) as well as public advertisement and collaborating audiologists in the surrounding areas of Berlin and Hamburg.

Based on sample size calculation (for details see section “Sample Size Estimate/Power Calculations”), a group of 96 community-dwelling older adults (50–70 years) and 48 younger adults (20–30 years) will be recruited. The older experimental group will be divided into the following 2 equally sized subgroups: (1) hearing impaired, (2) not hearing impaired. The participants’ allocation to the group will be done after prior assessment using a test battery in a first session. Data analyses will be done on anonymized data.

#### Confidentiality

All recorded data will be pseudonymized using a participants’ number. Only the individual participant code recreated by the participant will allow identifying the participants number of the respective data set in case data deletion is requested within thirty days after end of the data recording. No questionnaire or digital data file will include names or other personal information that would allow identification of data-participant-relations. Video recordings will be stored only after pixelating the face (e.g., Sensarea). Raw data of video will be already deleted permanently on the day of the recording as soon as the pixelating is done.

#### Eligibility Criteria

The group assignment will be based on the severity of hearing impairment using 4-frequency (0.5, 1, 2, and 4 kHz) pure-tone average (PTA 0.5–4 kHz) and defining PTA 0.5–4 kHz ≤ 25 dB HL as normal and PTA 2–4 kHz = 26–40 dB HL as mild in the better hearing ear. Participants will be asked to do the hearing acuity testing with and without their hearing aid to record if their hearing ability was corrected to normal which has effects on the sensory stimulation of the brain.

Inclusion criteria for the healthy young participants will be (1) no diagnosis of hearing impairment (PTA 0.5–4 kHz ≤ 25 dB HL), (2) age range between 20 and 30 years, (3) no color blindness.

Inclusion criteria for the non-hearing-impaired older participants will be: (1) no diagnosis of hearing impairment (PTA 0.5–4 kHz ≤ 25 dB HL), (2) score > 7 for the Short Physical Performance Battery (SPPB), (3) living independently in the community, (4) maximally moderate risk of falls (< = 13% in the QuickScreen Clinical Falls Assessment Tool), (5) age range between 50 and 70 years, (6) no color blindness.

Inclusion criteria for the hearing-impaired older participants will be: (1) diagnosis of mild hearing impairment (PTA 2–4 kHz = 26–41 dB HL), (2) score > 7 for the SPPB, (3) living independently in the community, (4) maximally moderate risk of falls (≤ 13% in the QuickScreen Clinical Falls Assessment Tool), (5) age range between 50 and 70 years, (6) no color blindness. The severity of hearing impairment will be based on the pure tone audiometry results (evaluated by qualified audiologists prior to the pre-screening session).

Exclusion criteria will be (1) severe hearing impairment (PTA 0.5–4 kHz ≥ 41 dB), (2) any acute or chronic diseases, especially of the peripheral and central nervous system, (3) recurrent falls, (4) impaired vision that is not corrected with e.g., glasses, (5) SPPB score ≤ 7, (6) indication for impaired cognition (MoCA), (7) risk of falls > 13% (QuickScreen Clinical Falls Assessment Tool).

### Outcome Measures

The assessment will focus on behavioral, gait-related as well as neurophysiological markers to gain a deeper understanding of the CMI during dual-task walking. Standardized questionnaires and assessments, as described in the section “Secondary Outcomes,” will be used to collect general health information and used to control for covarying factors.

#### Primary Outcomes

The primary outcome of the present study will be the dual-task cost occurring in over-ground walking with a cognitive secondary task. Gait parameters will be recorded for single and dual task walking, EEG measures will be conducted throughout the entire experiment (ST cognitive; ST walking; DT walking).

##### Cognitive Task Performance

Participants will receive an auditory or visual discrimination task which fits all criteria for the analyses of gait parameters and event-related as well as continuous brain activity. Task performance will be analyzed using response accuracy and response time.

##### Gait Performance

Gait parameters like walking speed, step length, double support time, etc., will be captured by the OptoGait system (Microgate, Italy). The OptoGait utilizes photoelectric bridges between LEDs and photodiodes to record ground contacts. Therefore, two parallel positioned rows of bars frame the area of measurement. The temporal resolution of the OptoGait is 1 kHz, with a spatial resolution of 1.041 cm. The OptoGait has already been cross-validated against a three-dimensional motion capture system (ICC 0.690–0.999; *p* < 0.001; Healy et al., 2019). The heel strike will be used as event to calculate gait parameters such as step length, swing phase, stance phase, double support phase and more. The heel strike has been proven to be the most suitable event for our purposes (Rudisch et al., 2021).

Arm swing and hip rotation will be recorded using the HTC Vive trackers and four lighthouse cameras (HTC Corporation, Taoyuan City, Taiwan). Both measures will be analyzed for regular and natural patterns using the amplitude and variability of the respective measure.

##### Brain Activity Changes

EEG activity will be recorded using 64 active electrodes on an elastic cap (actiCAP snap and LiveAmp 64, Brain Products GmBH, Gilching, Germany) with electrode positions of the 10% system (Oostenveld and Praamstra, 2001) and one electrooculography electrode placed on the cheek for capturing vertical eye movements. Brain activity data will be analyzed on the sensor- as well as source-level. Individual electrode positions will be recorded using a handheld scanner (CapTrak, Brain Products GmBH, Gilching, Germany).

Event-related measures of interest will be event-related potentials (ERPs, e.g., visual P1/N1, P3 over the occipital and parietal cortex, auditory P1/N2, P3 over the temporal and parietal cortex) as well as event-related spectral perturbations (ERSPs, visual and auditory evoked, and event-related desynchronization of mu (10–12 Hz) and beta (18–30 Hz) rhythms during walking). Further analyzed ERPs will be response-related slow cortical potentials (e.g., movement-related cortical potentials like the lateralized-readiness potential at left and right central electrode sites), and eye-movement as well as gait-phase related analysis. EEG data will be analyzed in the time and frequency domain. In addition, functional connectivity measures will be investigated.

#### Secondary Outcomes

Secondary outcome measures will be recorded in order to ensure the eligibility criteria and to control for the influence of various covarying factors.

##### Demographic and General Questionnaire Regarding Participants’ Current State

Demographic data such as age, body height and body mass, sex, and socio-educational status will be collected, as well as data on the current state of health on the respective assessment day. As a possible contributing factor to the impact of the response side (e.g., on reaction time in cognitive tasks), participants will be asked for their handedness in ten everyday life situations.

##### Short Falls-Efficacy-Scale-International

The Short Falls-Efficacy-Scale-International (SFES-I) is a 7-item questionnaire addressing fear of falling during easy and complex physical activities as well as social activities (Yardley et al., 2005). A validated German version is available, the completion time is approximately 10 min.

##### QuickScreen Clinical Falls Assessment Tool—Translated to the German Language

The QuickScreen Clinical Falls Risk Assessment is a multifactorial assessment tool adapted to clinical settings. Measurement properties have already been confirmed in cohorts of community-dwelling older adults. The QuickScreen shows low measurement error, good reliability, and high sensitivity for physical status changes (Tiedemann et al., 2010). The instrument captures information on risk factors of falling in about 10 min. Assessed risk factors are (1) previous falls, (2) medication usage, (3) vision, (4) peripheral sensation, (5) lower limb strength, (6) balance, and (7) coordination. The number of affirmed risk factors is translated into the potential risk of falling for the respective participant, expressed as a percentage. The calculated percentage indicates the risk of falling within the next year. Therefore, a higher value represents a higher risk of falling.

##### Montreal Cognitive Assessment (MoCA)

The Montreal Cognitive Assessment is a one-page 30-items test developed for screening Mild Cognitive Impairment. It involves items to assess a range of cognitive domains, including executive functions, visuospatial abilities, language, attention, working memory, abstraction, and orientation to time and place. The internal consistency of the MoCA is good (Cronbach’s alpha = 0.84; Wong et al., 2018) and a validated German version is available. The duration of the assessment is about 10 min.

##### Short Physical Performance Battery (SPPB)

The Short Physical Performance Battery (Guralnik et al., 1994) assesses the valid physical function of the lower extremity in older people. Participants are required to stand in an upright position under three conditions (Romberg stance, semi-tandem stance, tandem stance). After that, comfortable gait speed is assessed by measuring the time to walk a four-meter track, starting from a standing position and stopping when the first foot is at the four meters line. Finally, a five-time sit-to-stand transfer is completed as fast as possible. Each domain is scored between zero and four points; SPPB overall scores range from zero (low mobility) to twelve (full mobility). Participants with a score less than 8 will be excluded. The SPPB takes about 10 min.

##### Pure Tone Audiometry

Qualified audiologists with their medical-approved equipment will perform the audiometry on-site testing both ears of the participants. When hearing aids are used by the participant during everyday life, the measurement will be performed with and without a hearing aid. Special solutions for this situation will be conducted in cooperation with the hearing aid acoustician.

##### Dual-Task Strategy Assessment

Participants will be asked to answer a customized six yes/no-items questionnaire about the strategy used during dual-task walking with a modified version of the questionnaire used by Wollesen et al. (2017a):

*Did you feel insecure while walking with the addition of the visual task?*

*Did you feel insecure while walking with the addition of the auditory task?*

*I was annoyed by the mistakes I made in the secondary tasks.*

*Did you concentrate more on the secondary tasks compared to gait performance?*

*Did you try to equally allocate attention to walking and the secondary tasks?*

*Was your preferred walking speed slower while walking with a secondary task?*

*(German Translation: Haben Sie sich unsicher gefühlt, als Sie während des Laufens die Farbe der Lichtblitze unterscheiden sollten? Haben sie sich unsicher gefühlt als Sie während des Laufens die Tonhöhe der Töne unterscheiden sollten? Ich habe mich über meine Fehler in den Entscheidungsaufgaben geärgert. Haben Sie sich mehr auf die Entscheidungsaufgabe konzentriert als auf das Gehen? Haben Sie versucht Ihre Aufmerksamkeit gleichermaßen auf Gehen und Entscheidungsaufgabe zu verteilen? Hat die Entscheidungsaufgabe dazu geführt, dass sich Ihre präferierte Ganggeschwindigkeit verlangsamt hat?)*

### Procedure

#### Description of the Testing Procedure

The cross-sectional study will consist of 2 days of measurement for each participant. After recruitment and signing informed consent, participants’ characteristics will be gathered via a standardized assessment (Prescreening) on day one lasting approximately 1.5 h. Questionnaires will be utilized to capture demographic characteristics, health assessment, fear of falls, fall risk, and cognitive capacity. The physical function will be rated by leg strength, gait- and balance testing. Pure tone audiometry and familiarization with the measurement setup of day two will complete the first day of measurement. The utilized instruments are listed in chronicle order of application below:

##### Measurement Day 1: Prescreening to Identify Confounders and Security Risks

a) Demographic and general questionnaire regarding participants’ current state
b) Handedness Questionnaire
c) Short Falls Efficacy-Scale-International (SFES-I)
d) QuickScreen Clinical Falls Assessment Tool
e) Montreal Cognitive Assessment (MoCA)
f) Short Physical Performance Battery (SPPB)
g) Pure Tone Audiometry
h) Familiarization with the experimental setup

At the end of day one, participants fulfilling the requirements for inclusion in the study will be invited to return on another day for the second set of measurements. The measurements on day two will mainly comprise EEG measurements while sitting and walking under single- and dual-task conditions. The order of measurement conditions will be pseudo-randomized on day two, always starting with a walking condition and then constantly switching between walking and sitting conditions. Dependent on the task instructions, one of the combinations will represent motor-response ipsilateral (cyan-right, magenta-left, high pitch-right, low pitch-left) or contra-lateral (magenta-right, cyan-left, high pitch-left, low pitch-right) to the presentation side (Table 1). Following the questionnaire about the current state of the participant, familiarization trials for auditory and visual stimuli, and 3 min baseline recordings during sitting and walking will take place.

**TABLE 1:** Stimuli number of the cognitive tasks dissociated by task condition, presentation side, cognitive task modality, modality-specific properties, and presentation-response compatibility with shaded entries representing that the correct response is contralateral to presentation side.

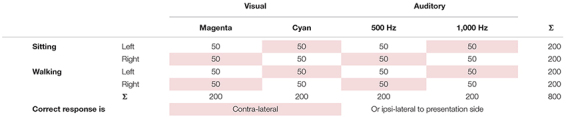	

##### Measurement Day 2: Dual-Task Walking With EEG

a) General questionnaire regarding participants’ current state
b) Baseline EEG and Gait Performance without response device
c) Single-Task-Walking with a response device
d) Cognitive tasks while sitting (Single-Task-Conditions)
e) Cognitive tasks while walking (Dual-Task-Conditions)

Measurement day 2 will take approximately 2.5–3.5 h per participant and will string together conditions of sitting and walking. Breaks will be at least 2 min between the conditions and may be extended as required. At the end of the measurement of day 2, the participants will be asked about their dual-task strategy.

#### Baseline Electroencephalography and Gait Performance Without Response Device

To collect EEG baseline data and to familiarize participants with the setting, participants will first sit quietly for 3 min and then walk at their preferred walking speed within a 10-m gangway of the OptoGait (Microgate, Bolzano, Italy) system without the response device (3 min). Following the baseline measurements, a 2 min break will take place.

#### Single-Task Walking With Response Device

Single-task walking will take place as one of five conditions in pseudo-randomized order. Participants will walk constantly 520 m (400 m within the OptoGait), but with the response devices in their hands. Participants will be able to choose their preferred walking speed and turn around by walking around a cone placed at least 1 m from the ends of the OptoGait gangway. From the single-task walking, we will also extract the distance walked within the first 6 min as a slightly modified measure of the 6-min walk test (ATS Committee on Proficiency Standards for Clinical Pulmonary Function Laboratories, 2002).

#### Cognitive Tasks While Sitting—Single-Task-Conditions

Participants will be asked to perform cognitive tasks (stimulus-discrimination). During these tasks, participants will be wearing the EEG-system, a custom-made spectacle frame with two LEDs attached to present light stimuli in the peripheral visual field. In addition, participants will wear headphones for presenting auditory stimuli. While sitting in the middle of the OptoGait and performing the task, participants will be asked to look in a walking direction. 200 stimuli (in either the visual or the auditory condition) will be presented with a varying inter-stimulus interval (400–800 ms) and a duration of 100 ms. Participants will be asked to react within a time window of 900 ms to the stimuli by pressing a button on the Vive controller in the respective hand. Thus, the maximal trial duration will vary between 1.4–1.8 s. The participant’s response will initiate the next trial and in case no response is given within the required time window, the next trial will start after 900 ms.

Visual task: Visual light stimuli, magenta (red and blue LED) and/or cyan (green and blue LED) flashes, will be presented counterbalanced and pseudo-randomized on the left or right side of the spectacle frame. Participants will have to which color (magenta or cyan) was presented (color discrimination task) with a right/left hand button press.

Auditory task: Auditory stimuli (high and low tone of 1000 and 500 Hz) will be presented for the duration of 100 ms either binaurally or to the left or right side with a volume of 50–60 dB (equals volume of an indoor conversation, Pearsons et al., 1977), regardless of presence and severity of the hearing impairment. Participants will have to indicate which pitch (low or high) was presented (pitch discrimination task) with a right/left hand button press.

#### Cognitive Tasks While Walking—Dual-Task-Conditions

For the dual-task conditions, a walking time of 6–7.5 min is estimated for each condition, including turnarounds at the end of the gangway. The same visual and auditory stimuli as described in the previous section will be used. Participants will be asked to simultaneously walk up and down the OptoGait gangway. Participants will be able to choose their preferred walking speed and turn around by walking around a cone, placed at least 1 m from the ends of the OptoGait gangway. Five stimuli will be presented each time participants walk the 10 m gangway of the OptoGait. Each task will end after the participants walked 520 m (400 m within the OptoGait) and 200 presented stimuli.

### Data Collection, Management, and Analysis

#### Data Collection

Both, single-task (ST) and dual-task (DT) will be investigated in separate conditions in the experiment. For this purpose, over ground walking will serve as the primary task, while secondary tasks comprise visual or auditory stimulus discrimination. The within-subject comparison between conditions will enable the investigation of dual-task costs vs. task-specific demands. Furthermore, a between-subject comparison will allow for comparing the performance of hearing-impaired older persons with healthy young and older adults. All groups will be treated identically to allow for a comparison of results of dual-task performance across groups.

#### Data Management

All data gathered by questionnaires will be digitalized for further processing. Functional testing will be controlled via Unity3D (Unity Technologies, San Francisco, United States), which also assigns basic demographic data to the measurement data of each participant. All functional measurements will be streamed and synchronized using the LabStreamingLayer (Swartz Center for Computational Neuroscience, UCSD, US). Synchronized data streams will comprise gait analyses (OptoGait, Microgate, Bolzano, IT), EEG recording (LiveAMP, Brain Products, Gilching, DE), kinematics (VivePro, HTC, Taoyuan, TW), and secondary task performance (VivePro, HTC, Taoyuan, TW & Raspberry Pi, Raspberry Pi Foundation, Cambridge, GB).

Data processing and feature extraction will be done using customized MATLAB scripts (The MathWorks, Inc.), EEGLAB (Delorme and Makeig, 2004). After importing all synchronized Motion Capture and EEG data streams, we will preprocess the EEG data using the bemobil-pipeline.^1^ This pipeline comprises filtering, data cleaning (channel and line noise), independent component analysis computation and equivalent dipole modeling for activity sources. Gait data will be analyzed separately as well as events will be extracted which in turn will be used in joint analyses like gait-phase-related brain activity changes. Following processing of the event-related potentials or spectral measures will be done with respective processing pipelines like the unfold-toolbox (Ehinger and Dimigen, 2019). Source-based analysis will apply repetitive k-means clustering to ensure a stable clustering solution for further analysis of clusters representing the brain regions of interest and connectivity measures.

#### Statistical Analysis

Descriptive data will be presented as mean (*M*) and standard deviation (*SD*). Normal distribution and homoscedasticity will be checked by the Shapiro-Wilk-Test and the Levene-Test, respectively. Dual-task costs will be analyzed using univariate analyses of variances (ANOVA) including the between-subject factors *age* (younger vs. older) and in case of older *hearing ability* (hearing impaired vs. non-hearing impaired) and the within-subject factors *task complexity* (single- vs. dual-task) and *stimulus modality* (visual vs. auditory).

Significance will be set at α = 0.05. The effect size will be presented as partial eta squared. In all *post hoc* comparisons we will control for multiple comparisons (e.g., Bonferroni). Statistical analyses will be done by using SPSS (IBM, SPSS Inc., Chicago, IL, United States) or R (R Core Team, 2013).

#### Sample Size Estimate/Power Calculations

A g^∗^power sample size calculation (*a priori*: *F*-tests; repeated measures, within-between interaction, effect size f = 0.25; alpha error prob = 0.05; power (1-beta error prob) = 0.8; number of groups = 3) revealed a total of *N* = 36 for each group. Due to an expected drop-out rate of about 20 percent, we will integrate at least 44 participants for each group. We aim to recruit 48 participants per group for the Prescreening.

## Discussion

This study aims to gain more insights into CMI while walking of older adults with hearing impairments by using a MoBI-approach. The analysis will be conducted to investigate whether older adults with and without hearing impairments as well as younger adults differ in their cognitive-motor performance while DT walking. Moreover, underlying processes of the interaction between motor and cognitive tasks will be identified at a behavioral and neurophysiological level comparing people with hearing impairments with healthy younger and older adults and while walking with an auditory or a visual secondary task. The overall hypothesis is that there are performance differences and corresponding neuronal correlates between the subgroups with the highest disadvantages for the group of hearing-impaired older adults. We hypothesize that the performance differences are linked to the different cognitive-motor processes; i.e., stimulus input, resource allocation, and movement execution. Moreover, for the different DT conditions (auditory vs. visual) we assume performance decrements within the auditory condition, especially for older, hearing-impaired adults.

As multitask performance mimics everyday life (Faulkner et al., 2007), an understanding of how we adapt to CMI is critical. For example, with increasing age, adults might need cognitive-motor strategies to reduce the risk of falling during daily walking situations (Lövdén et al., 2008; Godde and Voelcker-Rehage, 2010; Schaefer and Schumacher, 2011; Klotzbier et al., 2021). These strategies need to be tailored for adequate exercise interventions addressing different health-related problems, like hearing impairments, to overcome the CMI, for example in fall prevention. To gain more systematic and structured results of different aspects of cognitive-motor performance this study firstly addresses three blocks of behavioral and performance outcomes and their neurophysiological correlates.

Starting with the general characteristics of dual-task walking for all participants this study will compare the influence of visual or auditory secondary stimuli while walking. One might expect that with respect to age comparison the behavioral data might be different and that older adults show more changes in the observed gait parameters (e.g., speed, step length, double support time; refs). Because the walking performance between younger and older adults already differs at ST walking (Neider et al., 2011; Klotzbier et al., 2021), we expect especially the DT situation with additional visual input to lead to the highest performance decrements (for an overview cf. Beurskens and Bock, 2012). Additionally, there is first evidence that older and younger adults might show DT-related differences in walking patterns related to pace (like walking speed) or rhythm (like cadence or double support time; Beauchet et al., 2019; Klotzbier et al., 2021). The study by Klotzbier et al. (2021) showed that younger adults’ gait performance during a verbal fluency DT (naming animals) only led to reduced walking speed, whereas for the older adults both elements of pace and rhythm were affected (Klotzbier et al., 2021). It is of interest if these results could be replicated for the integrated visual and auditory tasks within this study. Moreover, we expect first insights into the neuronal correlates of the interaction of the secondary task and the two dimensions of gait performance (parameters of rhythm vs. parameters of pace and effects on arm swing as well as hip rotation) as well as the different phases of a gait cycle (gait initiation, swing phases as well as double support phase; for example as shown for persons with Parkinson’s disease; Fino et al., 2018).

Regarding the EEG, it is expected that during DT walking as compared to the single cognitive task, the early evoked P1 covaries in amplitude and latency with walking speed independent of age (Protzak et al., 2021). In addition, a reduction in the late positive complex in the dual-task setup as compared to the single task is expected only in young but not older participants (Protzak et al., 2021). However, older participants without hearing impairments might demonstrate less reduction in the late positive complex as compared to the hearing impaired group. Gait-related spectral modulations are expected in different frequency bands including alpha, beta, and gamma (Onton et al., 2005; Seeber et al., 2014; Wagner et al., 2016). Studies have shown that EEG spectral power in the μ and β band decreases over sensorimotor areas during walking on the treadmill (Severens et al., 2012) when compared to a static condition. The μ and β are suppressed during movement and their amplitudes are modulated locked to the gait-cycle phase during walking (Seeber et al., 2014). It remains unclear if this changes under DT conditions. Moreover, it needs to be investigated whether this also happens during over ground DT walking.

In addition, older participants with hearing impairments are expected to show reduced amplitudes in auditory evoked potentials compared to older participants with normal hearing and younger controls. Previous studies suggest that visual-evoked P1, N1, and P2 amplitudes might be significantly increased in hearing-impaired participants accompanied by increased activation of temporal areas underlying auditory processing. This suggests visual cross-modal reorganization (i.e., intact visual systems can recruit and repurpose deprived audio cortices for processing of their input (Campbell and Sharma, 2014). The described study within this protocol might have the potential to support this hypothesis while using two different sensory modalities and thus allowing for a direct comparison of the results within the same participants.

Findings will provide evidence of general mechanisms of CMI (ST vs. DT walking) as well as task-specific effects underlying changes in dual-task performance while over ground walking. We will use the newly acquired knowledge to tailor intervention programs on physical activity and falls prevention to the special needs of the target group of older adults with hearing impairments. Systematic assessment of individual dual-task performance compared to their healthy cohort, and gained insights into the neural-correlates of motor-control in different conditions will guide training interventions with the overall goal to reduce the number of falls within this target population.

## Ethics Statement

The study was approved by the Ethics Committee of the Department of Psychology and Ergonomics of the Technical University Berlin (registration number BPN_WOL_1_210129). Written informed consent will be obtained from all participants or their legal guardians before enrolment in the study according to the Declaration of Helsinki. Participation in the study is voluntary and there will be no inappropriate financial incentives. The consent form explicitly points to the voluntary character of participation as well as the option to terminate the experiment at any given time without consequences and without giving reasons. All participant information and data will be stored securely and identified by a coded ID number only to maintain participants’ confidentiality.

## Author Contributions

BW conceived of the study. AW with help of OV, MŠ, and BW drafted the manuscript. AW, OV, MŠ, MP, MF, KG, JP, UM, and BW initiated the study design and AW and OV helped with implementation. BW, UM, and KG were grant holders. All authors contributed to refinement of the study protocol and approved the final manuscript.

## Conflict of Interest

The authors declare that the research was conducted in the absence of any commercial or financial relationships that could be construed as a potential conflict of interest.

## Publisher’s Note

All claims expressed in this article are solely those of the authors and do not necessarily represent those of their affiliated organizations, or those of the publisher, the editors and the reviewers. Any product that may be evaluated in this article, or claim that may be made by its manufacturer, is not guaranteed or endorsed by the publisher.

## Footnotes

1
https://github.com/BeMoBIL/bemobil-pipeline

## Abbreviations

ADL: Activities of daily living
CMDT: Cognitive-motor dual-task
CMI: cognitive-motor interference
DT: Dual-task
DTC: Dual-task costs
EEG: electroencephalography
MoBI: Mobile Brain/Body Imaging
ST: Single-task.
